# Artificial Intelligence in Breast Cancer Screening and Diagnosis

**DOI:** 10.7759/cureus.30318

**Published:** 2022-10-15

**Authors:** Gayathri Dileep, Sanjeev G Gianchandani Gyani

**Affiliations:** 1 Department of Surgery, Jawaharlal Nehru Medical College, Datta Meghe Institute of Medical Sciences, Wardha, IND; 2 Department of Minimal Access and Robotic Surgery, Anglia Ruskin University, Chelmsford, GBR

**Keywords:** diagnosis, cancer, digital pathology, breast cancer diagnosis, breast cancer screening, artificial intelligence, breast cancer

## Abstract

Cancer is a disease that continues to plague our modern society. Among all types of cancer, breast cancer is now the most common type of cancer occurring in women worldwide. Various factors, including genetics, lifestyle, and the environment, have contributed to the rise in the prevalence of breast cancer among women of all socioeconomic strata. Therefore, proper screening for early diagnosis and treatment becomes a major factor when fighting the disease. Artificial intelligence (AI) continues to revolutionize various spheres of our lives with its numerous applications. Using AI in the existing screening process makes obtaining results even easier and more convenient. Faster, more accurate results are some of the benefits of AI methods in breast cancer screening. Nonetheless, there are many challenges in the process of the integration of AI that needs to be addressed systematically. The following is a review of the application of AI in breast cancer screening.

## Introduction and background

Breast cancer is one of the most pressing issues women of the 21st century face. It is a significant health problem [1] and the most frequently diagnosed cancer among women worldwide [2]. Many lives are lost due to breast cancer [3]. It has a great effect on the physical and mental health of women. Breast cancer is more effective to treat if diagnosed early. The effectiveness of treatment in later stages is poor [4]. Therefore, early diagnosis and prevention can be helpful in recording more cases and reducing the number of deaths. The incorporation of artificial intelligence (AI) into screening methods is a relatively new and emerging field that is very promising in the early detection of breast cancer, thus resulting in a better prognosis of the condition. Human intelligence has always triumphed over every other form of intelligence on this planet. The defining feature of human intelligence is the ability to use previous knowledge, adapt to new conditions, and identify meaning in patterns [5]. The success of AI lies in the capacity to reproduce the same abilities [6]. Digital pathology creates high-resolution images from tissue specimens mounted on glass slides and is of significance to the diagnostic field [7]. It transforms histology glass slides into digital images using computerized technology. It stores histologic information, which can later be used for the analysis of any other slide by allowing us to detect any variation from the normal and thereby evaluate the disease and its progression. This is extremely helpful in diagnosing various diseases, especially cancer [8]. The second highest number of deaths due to cancer in women is attributed to breast cancer [9]. The pathogenesis of breast cancer is complex, and its early diagnosis and control of disease progression can prove to be a herculean task in front of the medical fraternity [10]. Treatment regimens for breast cancer depend on the specific individual and more or less has to be tailor-made according to the patient's specificities. Technological advancements such as molecular imaging and genomic expression have enabled better cancer characterization. These techniques, coupled with previously existing surgical methods, lead to better results in treatment [11]. Over the last 10 years, AI microscopy has been used for handling highly magnified images for the analysis of abnormalities and it has given more precise results than manual methods. There has been tremendous progress in the last 50 years when it comes to the treatment modalities of breast cancer. Early diagnosis, less disability after treatment, and less severe side effects are some benefits acquired with modern technology [12]. The analysis of high volumes of data poses an obstacle to using AI systems. The ethical issues associated with this need to be discussed and resolved. An effort must be made to understand who would benefit from this technique to prevent unfair practices [13]. This article is based on various advancements made in the field of imaging, digital pathology, diagnosis, and treatment of breast cancer with the help of AI methods and the drawbacks associated with it.

Search methodology

This is a narrative review. The authors have collected information by browsing previously published articles on Google Scholar, PubMed, Sci-Hub, Elsevier, etc.

## Review

Digital pathology and cancer management

Digital pathology plays a vital role in modern medical practice. The availability of faster and cheaper options has made it easier for clinicians and pathologists to store and handle information. Advances in machine learning have enabled the combination of digital pathology and AI, offering image-based diagnosis possibilities [14]. Oncology especially is one of the fields that has benefited a lot from this advancement. The complex signaling and transcription pathways, connecting cancer, stromal, and immune cells can be displayed as images using this technology. The opportunity of viewing whole-sized images of tissue specimens has helped in the viewing of subvisual morphometric phenotypes and has made way for better patient care [15].

Analysis of data on pathological samples has become easier with digital pathology and has led to a more in-depth understanding of the data gathered. Digital methods allow data to be gathered, integrated, and analyzed to greater extents than what is achievable using traditional techniques, often with more efficiency than the latter. It has great potential of achieving more reliable, precise data of larger volume as outputs with a sizeable scale of summary and comparison [14]. Some ways in which AI algorithms work are described below. For AI algorithms, it is necessary to have good-quality training images. It is the work of the pathologist to manually identify and annotate the regions showing abnormalities or of any pathological significance. Under ideal conditions, it is performed by experts in the field [16]. Qualitative evaluation can quickly identify cell types accurately and give an insight into histological, morphological, and biologically relevant patterns. Qualitative analysis allows accessing data from tissue sides that are not possible to be evaluated manually. Manual methods have a higher margin of error in comparison to digital methods considering the errors in the accuracy of data collected and processed by humans may not be as precise as a computer run by AI [17].

Artificial intelligence and screening of breast cancer

Breast cancer is one of the leading causes of death among women across the world [18]. It is a very complex disease when it comes to pathogenesis, as there are a variety of factors like gender, heredity, genetics, environment, and occupation that predispose the individual to get the disease. The most effective way to tackle the disease is through early screening methods [19]. Breast cancer screening has markedly reduced mortality rates. The incorporation of AI into the screening methods such as the examination of biopsy slides enhances the treatment success rate. There has been an increased interest in this area over recent years, and the field seems to have a very promising future. Computational radiology is used in carrying out procedures that were previously carried out by experts, and it involves computer vision, lesion detection, or recognition of patterns for lesion detection for classification of lesions according to BIRADS (Breast Imaging Reporting and Data System) and systematic reporting (diagnosis). It also involves extracting imaging biomarkers for modeling therapy responses based on predictive and prognostic values. Machine learning and deep learning are some of the key aspects of AI required in breast cancer imaging. Machine learning is used to store a large dataset, which is later used to train prediction models and interpret generalizations [20]. Deep learning is the newest branch of machine learning and it works by establishing a system of artificial neural networks that can classify and recognize images [21]. AI in breast cancer screening primarily consists of object detection (segmentation) and tumor classification as benign or malignant [22,23].

**Figure 1:**
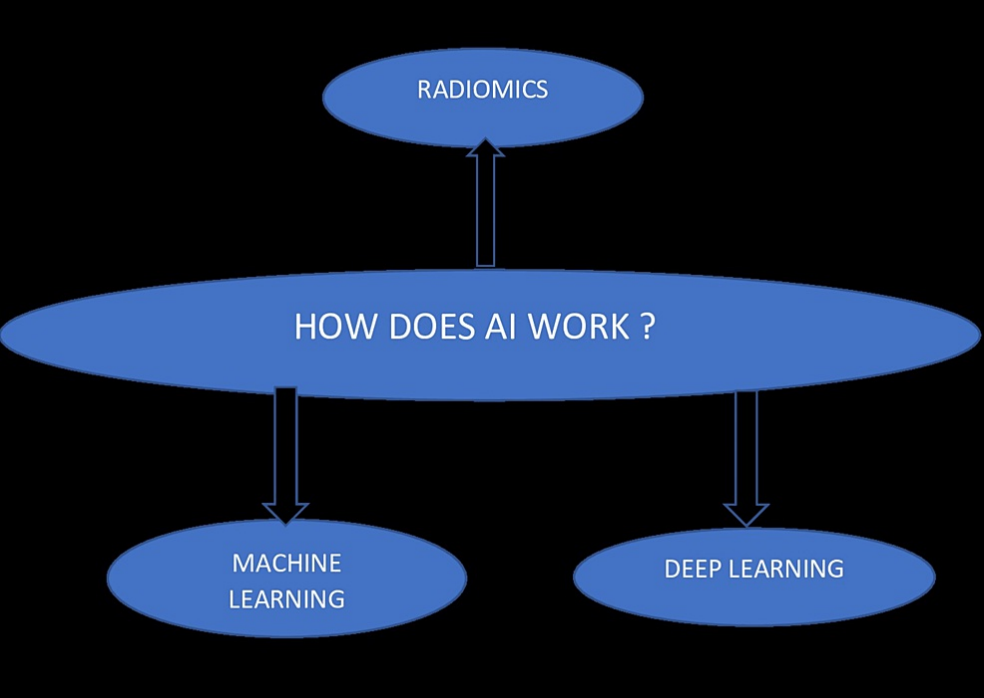
Mode of functioning of artificial intelligence

Radiomics is a technique widely used in AI systems. It extracts quantitative aspects from an image called a feature [24]. This usually occurs by pattern recognition algorithms that recognize images and provide as its outcome a set of numbers that represent a quantitative feature of the part of the image under view. Radiomics is based on the idea that extracted features represent various activities happening at the genetic and molecular levels. Machine learning consists of computational algorithms that make use of image features extracted by employing radiomics to help understand disease outcomes. There are two types of machine learning by radiomics: unsupervised machine learning and supervised machine learning. Unsupervised machine learning classifies information without the use of any pre-existing data or data obtained from the given image. The supervised machine learning method begins with AI training using a pre-existing data archive. Deep learning works by the processing of an image by a multi-neural layer or network, which reduces the image to a set of numbers that denotes features to be given as in the supervised machine learning method (Figure 1 and Table 1) [25].

**Table 1:** Different methods by which artificial intelligence works in breast cancer screening Adapted from [21].

Sr. No.	Method	Mode of functioning
1	Machine learning	A computational algorithm that makes use of image features
2	Deep learning	Processing of image by a multi-neural layer or network
3	Radiomics	Extracts quantitative aspects from an image, which is called a feature

In the field of breast cancer treatment, the use of AI for early detection by making use of data obtained by radiomics and biopsy slides is done. This is supported by a worldwide effort to manufacture learning algorithms for understanding mammograms by reducing the number of false positives as an outcome. AI has increased the odds of identifying metastatic breast cancer in whole slide images of lymph node biopsy. Because people's risk factors and predispositions differ, AI algorithms operate differently in different populations.

Mammography is the most popular method of breast cancer screening [26,27]. A high-resolution image is obtained, which is kept and further used without any limitation of age or body size. Full-field digital mammography systems have both input (raw images) and output (post-processing) formats. AI analyzes images and detects breast masses, mass segmentation, breast density, and cancer risk assessment. Breast masses are common findings in breast cancer patients, thereby making it one of the most important steps in computer-aided diagnosis (CAD) [28]. Calcifications appear as small spots on mammography and there are two in number: microcalcification and macrocalcification. At present, CAD systems are capable of detecting microcalcifications [29]. Breast mass segmentation, which is identified as true segmentation, directly affects the diagnosis. Fuzzy contours are used, which automatically segment breast masses from the mammogram. Breast segmentation is difficult to spot due to irregularities varying from one person to another. Proper segmentation using AI greatly improves the prognosis of the patient [30]. Breast density assessment is carried out using two-dimensional mammograms [31]. Breast cancer risk assessment is based on assessing risk factors such as age, family history, reproductive factors (menarche, menopause, age during the first pregnancy, parity, etc.), estrogen, and lifestyle of the individual [10,32].

Two decades ago, CAD became a part of screening mammography. Lots of studies were carried out to check the efficiency of single reading by radiologists versus double reading by CAD [32]. It did not necessarily show any advantage over the other, but the combination of both has reportedly shown a better success rate [33]. Studies have also shown that AI-based CAD has great scope to reach high sensitivity [34]. It can be used to reduce reading time in digital breast tomosynthesis (DBT) and can be used as a pre-screening tool for the exclusion of low-risk mammograms. CAD has been mostly used as an alternate opinion or decision support in patient care, but it must be subjected to proper scrutiny and demonstrate efficiency before integration. It is important to check the stability of results obtained over time [35].

Immunotherapy is a way by which we make use of the patient’s immune system responses in treatment. AI-based algorithms make it easy to detect neoantigens, but more investment is required in the field. AI can also be used to predict responses to immunotherapy. Studies have shown that links between immunotherapy response and radiomic characteristics bring to light uniform trends across all types of cancers and anatomical locations [36].

Challenges and future of artificial intelligence in breast cancer treatment

AI has become an extremely helpful tool in the field of cancer treatment. It has shown impressive outcomes and there arises a possibility that it could change every method of treatment currently. The only concerning question is where we draw the line between AI and human intelligence. AI is based on data collected from populations. Therefore, a disparity is sure to rise when it comes to the development of data on people belonging to different socioeconomic conditions [37,38]. Cancer is also one particular disease that has incidences that vary across different races. Studies related to the efficiency of AI have certain set outcomes that can be used to assess their standards and credibility. For AI machines to be truly accepted, people must be able to independently replicate and produce the machine like any other scientific finding. This implies a common code must be available to everyone, and it is only possible if data are shared with everyone equally [39].

AI models used for managing cancer are centered on image data. The problem with this particular aspect is the prevalent underutilization of patient histories saved as electronic health records in various hospitals. Easy-to-access databases and user-friendly software must be incorporated into the software systems of hospitals worldwide, which is a difficult task at the moment and necessitates the combined efforts of the medical and engineering communities [40,41]. Another challenge in the use of AI is building trust among doctors to make their decisions with the help of AI [42]. Adequate training must be provided to doctors on how to use AI technology [43].

In this modern era of mobile applications, it has become easier to retrieve data from people. Various parameters like blood pressure and heart rate can be monitored and easily obtained through these mobile apps. This has improved the quality of patient care and patient satisfaction. However, there are a lot of ethical risks to consider while using AI methods such as data confidentiality, privacy violation, the autonomy of patients, consent, etc. Many measures are taken to prevent any violation of confidentiality and legislation to keep a check on any malpractice [44]. Another limitation to using AI in breast cancer screening is that not much application of radiomics occurs in the present day-to-day clinical practice. Most of the studies conducted are also retrospective studies of small size, which make them less credible than large prospective studies. Nonetheless, AI would one day shortly possibly replace many roles carried out by a radiologist, and it is certain that if not replaced, it would aid the radiologist in coming to conclusions. Its noninvasive nature makes it a feasible option, and with further research, it would be able to harness more power of AI [25].

## Conclusions

Breast cancer has proven to be a great burden on the medical world and patients alike. The incorporation of AI into the different screening methods has made it easier to diagnose cancer early. Various ways in which AI works in breast cancer screening are machine learning, deep learning, and radiomics. These techniques are innovative and aid the pathologist in early diagnosis and quality patient care. However, the use of AI has its limitations. Many legislations are present to regulate the use of AI. AI can detect breast mass, segmentation, and density of breast tissue; furthermore, it can also identify calcification benefiting the diagnosis and management of patients. With further research and an upgrade in technology, it should be possible to overcome these challenges and make overall screening methods based on AI more popular, improving the overall quality of life in cancer patients.
